# Supplementary material for: Food taboos and preferences among adolescent girls, pregnant women, breastfeeding mothers, and children aged 6–23 months in Mainland Tanzania: A qualitative study
Source: PLOS Glob Public Health. 2024 Aug 12;4(8):e0003598. doi: 10.1371/journal.pgph.0003598 (PMC11318888; doi:10.1371/journal.pgph.0003598)
Supplement: S1 Text — (DOCX) [file pgph.0003598.s002.docx]

**S1 Text**

**FGD interview guide**

The participants should mention their numbers every time they respond to questions or provide inputs in the session.

Questions

1. What are the common foods consumed in this community *(Probe: How does it vary by season?)*
2. What kinds of foods are usually consumed by women like you specifically? *(Probe for the reasons for consumption of specific types of foods by women: religious beliefs, ethnicity, traditional beliefs, norms, availability, income/food prices, and others)*
3. What foods should women like you not eat? *(Probe for the reasons: What are specific beliefs and conceptions about permitted and encouraged food items in this community?)*
4. What do women usually eat (prefer) before, during, and after child birth*? (Probe for the reasons for consumption of specific types of foods by women: food choices, religious beliefs, ethnicity, traditional beliefs, norms, availability, income/food prices, and others)*

*Probe: food choices, restriction of some food items, role of family members, significant others, peers and influential people on food choices.*

*Probe: What foods are most important for them to consume at these times? (Probe for the reasons)*

*Probe: What foods should women not consume at these times? (Probe for the reasons)*

1. Whose role is it to ensure good nutrition for women? *(Probe: What is their role? What should they do to ensure women have good nutrition? What do they usually do?)*
2. In this community what efforts are being taken to ensure that women of reproductive age access and consume appropriate food items: *(Probe for existing interventions at local and national level)*
3. What kind of nutrition counseling and education is available, if any? *(Probe: Who gives them nutrition education [community health workers, health care providers—nurse or doctor]? What are they told during nutrition counseling and education?)*
4. What are the existing sources of information on appropriate food items to be consumed by women of reproductive age? Who do you prefer to go to for advice? *(Probe for issues being communicated and perceived strengths and weaknesses).*
5. What can be done to provide nutrition education for women appropriately? *(Probe for communication channels choices, models of information delivery, their strengths and weaknesses).*
