# Supplementary material for: Food taboos and preferences among adolescent girls, pregnant women, breastfeeding mothers, and children aged 6–23 months in Mainland Tanzania: A qualitative study
Source: PLOS Glob Public Health. 2024 Aug 12;4(8):e0003598. doi: 10.1371/journal.pgph.0003598 (PMC11318888; doi:10.1371/journal.pgph.0003598)
Supplement: S2 Text — (DOCX) [file pgph.0003598.s003.docx]

**S2 Text**

**In-Depth Interview Guide for Nutrition Officers**

Kindly, tell us about your age, education level, position, and duration in service [record gender as observed].

1. Can you briefly explain to me your roles as nutrition officer in this district/municipality?
2. What are the common foods consumed in this community? (Probe: How does it vary by season?)
3. What kinds of foods are usually consumed by women in particular? (Probe for the reasons for consumption of specific types of foods by women; religious beliefs, ethnicity, traditional beliefs, norms, availability, income/food prices, and others.)

*Probe: When they have the choice, what do women prefer to eat? What are the reasons for their preference?*

1. What kinds of foods are usually consumed by adolescent girls (15–25 years of age)? (Probe for the reasons for consumption of specific types of foods by adolescents: food choices, religious beliefs, ethnicity related, traditional beliefs, norms, availability, income/food prices, and others.)

*Probe: When they have the choice, what do adolescent girls prefer to eat? What are the reasons for their preference?*

1. How do women usually change their diets during pregnancy? (Probe for the reasons for consumption of specific types of foods by women; food choices, religious beliefs, ethnicity related, traditional beliefs, norms, availability, income/food prices, and others. How appropriate are these changes? Which ones should be perpetuated and why? Which ones should be changed and why?)
2. What foods are pregnant mothers supposed to eat? (Probe for the reasons. What are specific beliefs and conceptions about permitted and encouraged food items in this community?)

*Probe: What foods should pregnant women not eat? (Probe for the reasons. What are specific beliefs and conceptions about restricted food items in this community?)*

*Probe: What foods should women eat after pregnancy? (Probe for the reasons. What are specific beliefs and conceptions about permitted and encouraged food items in this community?)*

*Probe: What foods should women not eat after pregnancy? (Probe for the reasons. What are specific beliefs and conceptions about restricted food items in this community?)*

*Probe: What foods should women eat when breastfeeding? (Probe for the reasons. What are specific beliefs and conceptions about permitted and encouraged food items in this community?)*

*Probe: What foods should women not eat when breastfeeding? (Probe for the reasons. What are specific beliefs and conceptions about restricted food items in this community?)*

1. What foods are important to feed children under two years of age? (Probe for the reasons for consumption of specific types of foods by children; food choices, religious beliefs, ethnicity related, traditional beliefs, norms, availability, income/food prices, and others.) How appropriate are these foods for young children? Which ones should be perpetuated and why? Which ones should be changed and why?)

*Probe: What foods should children under two years not eat? (Probe for the reasons. What are specific beliefs and conceptions about restricted food items in this community?)*

1. In your family or community, what efforts do you take to ensure that women of reproductive age and children access and consume appropriate food items?

*Probe: What efforts are taken by others in the community? (Probe for existing interventions at local and national level.)*

1. What kind of nutrition counselling or education, if any, is available here? (Probe: Who gives them nutrition education [community health workers, health care providers—nurse or doctor]. What are they told during nutrition counselling and education?)

*Probe: What are the existing sources of information on appropriate food items to be consumed by women of reproductive age and children? (Probe: issues being communicated and perceived strengths and weaknesses).*

10. What can be done to provide nutrition education for women and children appropriately? (Probe: communication channels choices, models of information delivery, their strengths and weaknesses).
