# Supplementary material for: Food taboos and preferences among adolescent girls, pregnant women, breastfeeding mothers, and children aged 6–23 months in Mainland Tanzania: A qualitative study
Source: PLOS Glob Public Health. 2024 Aug 12;4(8):e0003598. doi: 10.1371/journal.pgph.0003598 (PMC11318888; doi:10.1371/journal.pgph.0003598)
Supplement: Attachment — Submitted filename: A rebuttal letter_08_07_2024.docx [file pgph.0003598.s004.docx]

Dear Hasanain Faisal Ghazi, PhD

Thank you very much for giving us the opportunity to submit a revised draft of our manuscript titled “Food taboos and preferences among adolescent girls, pregnant women, breastfeeding mothers and children of 6-23 months in Mainland Tanzania”
We appreciate the time and effort that you and reviewers have dedicated to providing valuable feedback on our manuscript.

Here is a point-by-point response to the reviewers’ comments and concerns.

**Review Comments to the Author**

**Reviewer #1**

**Comment 1**: The title is descriptive and clearly outlines the focus and scope of the study. However, it could be improved for clarity. Here is a revised version: " Food taboos and preferences among adolescent girls, pregnant women, breastfeeding mothers, and children aged 6-23 months in Mainland Tanzania" I added "aged" before "6-23 months" to make it clearer that the authors are referring to the age range of the children. Additionally, I added a comma after "breastfeeding mothers" to maintain parallel structure with the rest of the list. This may be considered.
**Response:** Thank you very much and, we agreed with your insightful suggestions and re-write the title as suggested (see page 1).

**Comment 2:** The introduction provides insight into the study's context and rationale. However, to enhance clarity, authors may consider incorporating a paragraph elucidating the theoretical definition of food taboos, food restrictions, and food preferences. Furthermore, augmenting the discussion with relevant literature on how these taboos and restrictions, and preferences shape diet related behaviours that may have long-term consequences for the health and nutrition of a population.

**Response:** Thank you very much and we agree with your comment. We have re-write the introduction section incorporating a paragraph elucidating the theoretical definition of food taboos, food restrictions, and food preferences (page 4, paragraph 2) and augmenting the discussion with relevant literature (see page 4-6).

**Comment 3**: The concluding sentences in the introduction (The findings of this study will provide information to help improve vital maternal and adolescent nutrition interventions in Tanzania. The insights gained to support the prevention and management of micronutrient deficiencies and integrated management of acute malnutrition by providing education on food taboos and preferences that may affect maternal and adolescent nutrition) are very generic and vague. It is essential for the authors to contextualize the significance of understanding prevalent food taboos/restrictions/preferences within the specific scope of this research. By explicitly stating how this understanding of food taboos/restrictions will contribute in addressing the malnutrition situation in Tanzania. This could involve highlighting how insights into food taboos/preferences among adolescent girls, pregnant women, breastfeeding women, and young children aged 6-23 months will inform targeted educational interventions aimed at improving dietary practices and nutritional outcomes

**Response:** Thank you very much and, we have re-write the concluding sentences as suggested (see page 6, paragraph 2).

**Comment 4**: Methods
Data analysis: Please also mention whether you utilised any software for data analysis, such as ATLAS.ti, NVivo etc. (if any).

**Response:** Thank you very much and we agree with your comment. We added the software used for data analysis (see page 8 paragraph 3).

**Comment 5**: Ethical consideration: Please mention the ethics training (if any) given to the research team for empowering them to: recognize power dynamics and its potential influence; and reflect on personal biases and assumptions during the process of data collection.

**Response:** Thank you very much and we agree with your comment. We have incorporated the suggestion (see page 10 paragraph 1).

**Comment 6**: Results
The results shed light on the dietary preferences/restrictions of the demographic groups which are under study. The data presented in the paper is more about the prohibition/restriction of certain foods during pregnancy and which is explained as being aimed at protecting the health of both the mother and the baby. There is only limited information on food taboos which are based on misconceptions or misinformation, such as the belief that consuming eggs can result in a child being born without hair.
Most of the dietary restrictions reported by pregnant women are align with healthy practices, serving as protective measures against potential health complications. The question arises is that whether these dietary practices (nutrition information), encompassing avoidance of alcohol, cigarettes, soft drinks, and leftover food during pregnancy, can be classified as food taboos? If the answer is yes, how this will help in developing educational interventions aimed at improving optimal dietary practices and nutritional outcomes.

**Response:** We agree with your comment and we have rephrased the section as suggested (see page 10 paragraph 2).

**Comment 7**: The section ‘gap in nutrition awareness’ is seems like a discussion and appears not supported by the data from this research.

**Response:** We agree with your comment and we have deleted ‘section on gap in nutrition awareness’ as suggested (see page 18-19).

**Comment 8**: The statements such as ‘adolescents have limited understanding on appropriate eating practices and peer pressure usually drives their food preferences’ needs to be supplemented by verbatim.

**Response:** Thank you very much and we agree with your comment. We have incorporated the suggestion (see page 17).

**Comment 9**: The results section requires a more robust presentation of data, particularly emphasizing taboos rooted in misconceptions that significantly impact nutrition and health outcomes. A persuasive restructuring is necessary to ensure a coherent flow, supported by empirical evidence within each domain of investigation.

**Response:** Thank you very much and, we have re-write the results section as suggested (see page 10-19).

**Comment 10**: Discussion
In the discussion, the authors note that ‘fibres and fruits were not preferred, only postpartum and lactating mothers reported the consumption of fruits. These findings are similar to other studies that show low intake of fruits and vegetables among pregnant women, lactating women, adolescent girls, and children especially in low- and middle-income countries.' While the current research aims to explore food taboos, preferences, and restrictions, the consumption of fruits, vegetables, milk, etc. is largely influenced by individuals' purchasing power to incorporating them into their regular diet. This low intake is primarily due to economic constraints rather than behavioural factors

**Response:** **T**hank you very much and, we have re-phrased the sentence as suggested (see page 24).

**Comment 11**: Limitation: The study focused on specific demographic groups (adolescent girls, pregnant women, breastfeeding mothers, and children aged 6-23 months), potentially overlooking other pertinent stakeholders like adult men/women or elderly individuals, who also play roles in shaping food practices within households. Including these people in the research could have provided deeper insights into the subtleties of food taboos and their cultural underpinnings. Understanding the belief systems and misconceptions surrounding these taboos is essential for designing health programs that effectively address them through targeted health messaging.

**Response:** Thank you very much and we agree with your comment. We have incorporated the suggestion (see page 19).

**Reviewer #2
Comment 1:** Title
1. It is highly recommended to indicate the nature and topic of the study identifying the study as qualitative or indicating the approach of ethnography in the title.
**Response:** Thank you very much and we agree with your comment. We have rephrased the title as suggested (see page 1).

**Comment 2**: Introduction
1. “In Tanzania, a study conducted in the southern part of the country shows about two thirds of the women avoided fish and farm meats [18] and, in the northern part of the country, pregnant women are restricted from consuming unpasteurized milk, meat, or milk from cattle (other than their own), eggs, sweet foods, and butter for preventing complications during delivery, especially associated with the delivery of a large baby [19]. “
Please check if the reference [18] reported this result. In this study, the cross-sectional descriptive study design can only show the proportion of dietary intake of food, but not if a women avoid. The authors should be careful when describing findings of previous studies throughout the text.
In addition to the previous study cited as reference [19], what is already known from previous studies? Please provide a brief summary of existing evidence from literature and clarify the literature gap that the authors addressed in this study..

**Response**: Thank you very much for pointing out this and, we have carefully described the findings of previous studies (see page 5-6)

**Comment 3**:

2. Are there any relevant theory regarding food taboo and preference in Tanzania or other similar cultures? This could help to specify or state research questions.
**Response**: We agreed with this suggestion and added a sentence as suggested (see page 4 paragraph 2).

**Comment 4**: Methods
1. Provide guiding theory if appropriate, and rationale for the ethnography method.

**Response:** Thank you very much and we agreed with this comment and added the rationale for the ethnography method (see page 4 and page 7).

**Comment 5**:

2. Provide researchers’ characteristics that may influence the research such as
personal attributes, qualifications/experience, relationship with participants, or assumptions; potential or actual interaction between researchers’ characteristics and the research questions, approach, methods, results, and/or transferability.

**Response**: We agree with your comment and rephrased the ethics section to incorporate the suggestion (See page 10).

**Comment 6**:
3. Provide information on criteria for deciding when no further sampling was necessary
(e.g., sampling saturation).

**Response:** Thank you very much and we have added more elaboration ‘sampling saturation’ as suggested (see page 8-9).

**Comment 7**:
4. Did the authors use other techniques to enhance trustworthiness and credibility of data analysis, such as audit trail, in addition to member check? If yes, please provide relevant information.

**Response:** Thank you very much for pointing out this and, yes we used audit trial (see page 9, paragraph 2)

**Comment 8**: Discussion
1. Trustworthiness and limitations of findings should be considered.

**Response:** Thank you very much and we agree with your comment. We have incorporated the suggestion (see page 19).

Sincerely,

Ray Masumo, PhD
